# Postoperative management of antithrombotic medication in microvascular head and neck reconstruction: a comparative analysis of unfractionated and low-molecular-weight heparin

**DOI:** 10.1007/s00405-020-06219-w

**Published:** 2020-07-24

**Authors:** Matti Sievert, Miguel Goncalves, Rosalie Tamse, Sarina K. Mueller, Michael Koch, Antoniu-Oreste Gostian, Heinrich Iro, Claudia Scherl

**Affiliations:** Department of Otorhinolaryngology, Head and Neck Surgery, Friedrich Alexander University of Erlangen-Nuremberg, Waldstrasse 1, 91054 Erlangen, Germany

**Keywords:** Free-flap reconstruction, Flap failure, Thrombosis, Head and neck, Antithrombotic agent

## Abstract

**Purpose:**

Free flap reconstruction is a valuable technique to preserve function in oncological head and neck surgery. Postoperative graft thrombosis is a dreaded risk. This study aims to compare low-dose unfractionated heparin (UFH) and low-molecular-weight heparin (LMWH) in perioperative thrombosis prophylaxis.

**Methods:**

This is a retrospective analysis of 266 free flaps performed at our academic center. A comparison was made between 2 patient groups, based on their respective postoperative prophylaxis protocols either with UFH (*n* = 87) or LMWH (*n* = 179). Primary endpoints were the frequency of transplant thrombosis and the number of flap failures. Secondary endpoints were the occurrence of peri- and postoperative complications.

**Results:**

The flap survival rate was 96.6% and 93.3% for the groups UFH and LMWH, respectively (*P *= 0.280). The rate of postoperative bleeding requiring revision was 4.6% and 6.7% for each group, respectively (*P* = 0.498). We found a hematoma formation in 4.6% and 3.9% (*P* = 0.792).

**Conclusion:**

The free-flap survival rate using low-dose UFH seems to be equivalent to LMWH regimens without compromising the postoperative outcome. Consequently, for risk-adapted thrombosis prophylaxis, either LMWH or UFH can be administrated.

## Introduction

Microsurgical free-flap reconstruction following ablative surgery is a frequently used procedure for a large resection volume and maintains the functional outcome in patients with head and neck cancer [1]. The opportunity for free tissue transfer has many advantages over pedicled flaps if primary wound closure is not possible. A maintained volume leads to a better function of speech and swallowing [2, 3]. The outcome is dependent on the survival of the graft and proper integration into the adjacent tissue. The thrombosis-related reduced perfusion of the flap with consecutive necrosis of the tissue is a dreaded complication and a severe surgical emergency that necessitates immediate revision [1]. The majority of complications are attributed to thrombosis of the venous drainage [4]. In general, thromboses of the vascular pedicle are reported in up to 10% of micro anastomoses [5]. To prevent flap thrombosis, the use of anticoagulants appears to increase flap survival, which has been the objective of recent studies [6–8]. Heparin, dextran, and aspirin are often used anticoagulants in the management of microvascular reconstruction. Unfractionated heparin (UFH) is applied intravenously and inhibits circulating thrombin. Thrombin converts fibrinogen to fibrin, which leads to the formation of cross-linked fibrin, promotes platelet aggregation, and activates blood clotting factors V and VIII [9]. Low-molecular-weight heparin (LMWH) has an inhibitory effect on activated factor X. However, anti-thrombin activity is less intense [9]. Observations in animal studies showed the ability of LMWH to reduce thrombosis formation in microvascular anastomoses [10]. Few clinical studies are suggesting an improvement in outcome [11]. However, the superiority of a therapy regimen is still not evident [9, 11, 12]. The purpose of this study was to distinguish the difference between flap survival and postoperative complications depending on the use of intravenous low-dose UFH and subcutaneous LMWH in patients undergoing free-flap reconstruction in the head and neck area, to provide a recommendation for clinical practice.

## Materials and methods

### Study design

We conducted this retrospective study at a tertiary hospital and academic cancer center. Approval was given by the local institutional ethics committee and carried out following the Declaration of Helsinki.

### Eligibility criteria

We included all patients with ablative surgical procedures and microvascular flap transplantation, receiving postoperative antithrombotic therapy either with low-dose UFH (500 IU/h) or LMWH (20/40 mg daily) at our Department between January 2004 and July 2017. Patients who did not receive the low-dose UFH or LMWH protocol, for example, due to cardiac risk factors, or UFH/LMWH in therapeutic dosage, were excluded from the study.

### Characteristics of patient outcome

Patient information, which includes epidemiological, oncological, and operation-specific parameters, was recorded and analyzed retrospectively. Additionally, ASA Classification and patient-specific medical history were recorded to determine health conditions (Table 1). Furthermore, medical charts were reviewed for postoperative complications, such as partial or total flap failure, and minor or major complications like postoperative bleeding, hematoma formation, and salivary fistula (Table 2). Besides, we recorded thromboembolic events, such as deep vein thrombosis and pulmonary embolism.

**Table 1:** Patients’ characteristics for all patients and groups of patients divided based on postoperative thrombosis prophylaxis

	Group UFH (*n* = 87)	Group LMWH (*n* = 179)	All patients (*n* = 266)	Statistical comparison of groups UFH vs. LMWH *P* value
Gender (*n*, %)				
Male	72 (82.8%)	138 (77.1%)	210 (78.9%)	0.288
Female	15 (17.2%)	41 (22.9%)	56 (21.1%)	
Age (mean years ± SD)	57.9 ± 11.2	59.7 ± 10.5	59.1 ± 10.8	0.202
Tumor stage (*n*, %)^a^	(*n* = 82)	(*n* = 174)	(*n* = 256)	
T1	11 (13.4%)	16 (9.2%)	27 (10.5%)	0.317
T2	23 (28.1%)	68 (39%)	91 (35.5%)	
T3	28 (34.1%)	49 (28.2%)	77 (30.1%)	
T4	20 (24.4%)	41 (23.6%)	61 (23.9%)	
Location of primary (*n*, %)				
Oral cavity	16 (18.4%)	27 (15.1%)	43 (16.2%)	0.443
Oropharynx	41 (47.1%)	95 (53.1%)	136 (51.1%)	
Hypopharynx	16 (18.4%)	24 (13.4%)	40 (15%)	
Larynx	3 (3.4%)	14 (7.8%)	17 (6.4%)	
Other	11 (12.6%)	19 (10.6%)	30 (11.3%)	
Salvage surgery (*n*, %)	6 (6.9%)	14 (7.8%)	20 (7.5%)	0.788
Radiation dose in Gy, (mean ± SD)	69.9 ± 20.7	62.4 ± 13.6	65 ± 16	0.244
Flap type (*n*, %)				
Radial forearm flap	74 (85.1%)	119 (66.5%)	193 (72.6%)	0.002
Anterior lateral thigh flap	10 (11.5%)	57 (31.8%)	67 (25.2%)	
Other^b^	3 (3.4%)	3 (1.7%)	6 (2.3%)	
Venous anastomoses (*n*, %)^c^	(*n* = 85)	(*n* = 177)	(*n* = 262)	
1	37 (43.5%)	129 (72.9%)	166 (63.4%)	0.000
2	48 (56.5%)	40 (22.6%)	88 (33.6%)	
3	0 (0%)	8 (4.5%)	8 (3%)	
Use of coupler (*n*, %)	72 (82.8%)	146 (81.6%)	218 (81.9%)	0.485
Operating time (mean min. ± SD)	716 ± 217	674 ± 174	687.3 ± 189.6	0.117
Hospitalization (mean days ± SD)	21.3 ± 9.3	20.1 ± 16.3	20.7 ± 14.2	0.635
Time on ICU (mean days ± SD)	5 ± 2.6	4.3 ± 1.9	4.6 ± 2.2	0.189
Alcohol	34 (35.8%)	73 (40.8%)	107 (39%)	0.449
Smoking	75 (78.9%)	143 (79.9%)	218 (79.6%)	0.582
Pack years (mean ± SD)	37 ± 20	36 ± 18	36 ± 19	0.710
ASA-score^d^	(*n* = 84)	(*n* = 177)	(*n* = 261)	
1	2 (2.4%)	8 (4.5%)	10 (3.8%)	0.250
2	52 (61.9%)	112 (63.3%)	164 (62.8%)	
3	38 (45.3%)	57 (32.2%)	95 (36.4%)	
Heart failure (*n*, %)				
NYHA I	1 (1.1%)	3 (1.7%)	4 (1.5%)	0.026
NYHA II	9 (10.3%)	9 (5%)	18 (6.8%)	
NYHA III	6 (6.9%)	2 (1.1%)	8 (3%)	
PAOD (*n*, %)				
Fontaine 1	3 (3.5%)	3 (1.7%)	6 (2.3%)	0.797
Fontaine 2	0 (0%)	1 (0.6%)	1 (0.4%)	
Fontaine 3	1 (1.1%)	1 (0.6%)	2 (0.7%)	
Coronary heart disease (*n*, %)	10 (11.5%)	14 (7.8%)	24 (9%)	0.298
Apoplexy, c.a. (*n*, %)	6 (11.5%)	11 (6.1%)	17 (6.4%)	0.759
Pulmonary diseases (*n*, %)	11 (12.6%)	18 (10.1%)	28 (10.5%)	0.484
Diabetes mellitus (*n*, %)	11 (12.6%)	22 (12.3%)	33 (12.4%)	0.881
Hypertonia (*n*, %)	37 (42.5%)	77 (43%)	114 (42.9%)	0.937

*UFH* unfractionated heparin, *LMWH* low-molecular-weight heparin, *ICU* intermediate care unit, *ASA* American Society of Anesthesiologists, *NYHA* New York Heart Association, *PAOD* peripheral artery occlusive disease

^a^Data from 256 patients

^b^Latissimus dorsi and parascapular flap

^c^Data from 262 patients

^d^Data from 261 patients

**Table 2:** Patients’ outcome for all patients and groups of patients divided based on postoperative thrombosis prophylaxis

	Group UFH (*n* = 87)	Group LMWH (*n* = 179)	All patients (*n* = 266)	Statistical comparison of groups UFH vs. LMWH *P* value
Flap thrombosis (*n*, %)				
Total rate	8 (9.2%)	17 (9.5%)	25 (9.4%)	0.767
Arterial	3 (3.5%)	2 (1.1%)	5 (1.9%)	0.040
Venous	5 (5.7%)	15 (8.4%)	20 (7.5%)	0.445
Time to thrombosis (mean h ± SD)	29.8 ± 66.5	63.9 ± 100.7	57.5 ± 86.4	0.227
Total flap loss (*n*, %)	3 (3.4%)	12 (6.7%)	15 (5.6%)	0.280
Partial flap loss (*n*, %)	5 (5.7%)	4 (2.2%)	9 (3.4%)	0.134
Postoperative bleeding (*n*, %)				
Minor	3 (3.4%)	8 (4.5%)	11 (4.1%)	0.688
Major	4 (4.6%)	12 (6.7%)	16 (6.0%)	0.498
Hematoma formation (*n*, %)				
Minor	12 (13.8%)	22 (12.3%)	34 (12.8%)	0.731
Major	4 (4.6%)	7 (3.9%)	11 (4.1%)	0.792
Salivary fistula (*n*, %)				
Minor	9 (10.3%)	22 (12.3%)	31 (11.6%)	0.632
Major	4 (4.6%)	12 (6.7%)	16 (6.0%)	0.498
Overall revision rate (*n*, %)	18 (20.7%)	36 (20.1%)	54 (20.3%)	0.912
Deep vein thrombosis (*n*, %)	–	–	–	–
Pulmonary artery embolism (*n*, %)	1 (1.1%)	0 (0%)	1 (0.4%)	–
Heparin-induced thrombocytopenia	–	–	–	–
5-year-OS	66.7%	70.1%	69.0%	0.420
Follow-up (mean months ± SD)	58.6 ± 39.1	68.8 ± 43.6	65.4 ± 42.3	0.068

*UFH* unfractionated heparin, *LMWH* low-molecular-weight heparin, *OS* overall survival

### Characteristics of thrombosis prophylaxis

We matched all patients into two groups based on the type of postoperative, antithrombotic prophylaxis. Patients who received low-dose UFH after surgery, in the following, referred to as “group UFH”. In contrast, patients who received subcutaneous LMWH after surgery referred to as “group LMWH”. The surgeon decided due to the personal reference whether to apply UFH or LMWH. Thus, patient selection was not randomized. The intravenous application of low-dose UFH provided using a standard protocol with 500 IU per h via permanent infusion. By intention, the dosage was not administered to affect the activated partial thromboplastin time (APTT). All patients with LMWH received a standard protocol using enoxaparin (Clexane, Sanofi Aventis, Frankfurt, Germany). We assessed the individual risk of thrombosis based on exposure and dispositional risk factors, according to the Conference on Antithrombotic and Thrombolytic Therapy [13]. Patients with low peri- and postoperative thrombosis risk received 20-mg enoxaparin once a day. Patients with medium to high thrombosis risk (e.g., obesity) received 40 mg of enoxaparin once a day, according to the recommendations published [14]. Antithrombotic therapy was continued in both groups until the fifth postoperative day. We aimed for patient mobilization on the first postoperative day. Furthermore, we did not use other anticoagulants that influence on the coagulation time. As an indicator of coagulation, the APTT and the Prothrombin time, according to the Quick value (PT), were measured on day one to three (POD1–3), on day three to five (POD3–5) and on day eight to 12 after surgery (POD8–12) in each group (Table 3). For each group (UFH and LMWH), the coagulation parameters were compared at different time points to determine a change of coagulation or an overdose of heparin. It must be consciously mentioned that LMWH does not influence either APTT or PT.

**Table 3:** Coagulation parameters for all patients and groups of patients divided based on postoperative thrombosis prophylaxis

	Group UFH (*n* = 87)	Group LMWH (*n* = 179)	All patients (*n* = 266)	Statistical comparison of groups UFH vs. LMWH *P* value
APTT (mean ± SD)				
Preoperative	31.1 ± 4.4	32.1 ± 7.3	31.8 ± 6.1	0.261
POD 1–3	42.3 ± 12.6	38.6 ± 10.3	42.6 ± 15.1	0.077
POD 3–5	42.4 ± 26.2	35.9 ± 5.8	39.8 ± 18.5	0.092
POD 8–12	37.2 ± 9.6	36.2 ± 9.1	38.1 ± 10.5	0.633
PT (mean ± SD)				
Preoperative	91.2 ± 10.4	94.4 ± 9.5	93.6 ± 9.9	0.095
POD 1–3	74.3 ± 12.7	78.5 ± 13.3	76.2 ± 13.1	0.079
POD 3–5	83.7 ± 13.3	86 ± 12.4	84.8 ± 12.7	0.355
POD 8–12	79.6 ± 19.6	80 ± 20.2	79.8 ± 19.8	0.914

*UFH* unfractionated heparin, *LMWH* low-molecular-weight heparin; *APTT* activated partial thromboplastin time, *POD* postoperative day, *PT* prothrombin time

### Characteristics of microvascular anastomosis

We carried out all operations in a team of several surgeons from our ENT department, in which all members were qualified for the individual surgical steps. Therefore, each surgeon could alternately perform the tumor resection and neck dissections while the other surgeon performed the defect reconstruction. For time-saving reasons, the flap was harvested in parallel once the extent of the resection defect was determined. The surgeon, with the most experience, was responsible for performing the microvascular anastomosis. The arterial anastomosis was performed under a microscope in a single suture technique with 8.0 or 9.0 sutures. We performed the venous anastomosis either with a coupler device (Synovis, Micro Companies Alliance, Birmingham, United States of America) in end-to-end technique or with single sutures in end-to-end or end-to-side technique on cervical veins. Intraluminal heparin application was used in both groups during surgery when performing a microvascular anastomosis. All patients were monitored in the intermediate care unit in the first 48 h after surgery, with the head maintained in a 30° upright position to avoid compression of the neck. To prevent pressure to the neck region, we fixed the tracheal cannula with sutures. Flap surveillance was performed at a 2-h interval during the first 5 days by an experienced and trained ENT resident supervised by the microvascular surgeon. The flaps were monitored by clinical assessment of color and consistency as well as by Doppler ultrasound examination of the pedicle. The position of the pedicle had been marked intraoperatively by the ENT surgeon, who sutured the anastomosis. We defined flap failure as the interruption of arterial flow by observing pale skin or venous congestion by observing bluish-livid flap color, leading to surgical revision.

### Outcome parameters

Primary endpoints were the total frequency of transplant thrombosis and the number of flap failures. We defined total flap loss due to complete necrosis of the graft. In the case of partial flap loss, partial necrosis of the graft was without the restriction of functionality and continuity. Secondary endpoints were the occurrence of peri- and postoperative complications. Minor complications are defined by sufficient conservative therapy, while major complications, by definition, require surgical revision.

### Statistical analysis

Patients’ characteristics, as well as time values and radiation dose, are presented in mean and standard deviation (SD). Frequencies of oncological parameters and treatment modality are presented in absolute and relative values. The Chi square test performed the comparison of nominal parameters between group UFH and LMWH to show homogeneity between both groups. For the comparison of metric parameters between the two groups, we used the *t* test. Survival rates were calculated using the Kaplan–Meier method and compared by the log-rank test. The overall survival time was calculated from the date of surgery to the date of death from any cause or the date the patient was last known to be alive. We performed a binary logistic regression analysis to determine the influence of confounding variables on vascular pedicle thrombosis. A p-value of less than *P* ≤ 0.05 was considered statistically significant. For statistical analyses, we used SPSS Statistics (IBM Corp. Released 2013. IBM SPSS Statistics for Windows, Version 25.0. Armonk, New York, United States of America).

## Results

### Characteristics of subgroups

All patients’ and operational characteristics are presented in Table 1. We included a total of 266 patients undergoing free-flap reconstructions in this study. The groups UFH and LMWH compromised 87 and 179 patients, respectively. Group UFH included 72 men and 15 women (average age 57.9 ± 11.2 years). Group LMWH included 138 men and 41 women (average age 59.7 ± 10.5 years). All patients underwent free-flap reconstruction and uni- or bilateral neck dissection after oncological tumor resection. In both groups, there was an equal distribution of the T stage (*P* = 0.317) and the localization of the primary tumor (*P* = 0.443). In most cases (89%), free-flap reconstruction was used to restore the upper digestive tract. Remaining cases relate to the reconstruction of external defects. In the UFH group, a radial forearm flap (RFF) was performed in 85%, and an anterior lateral thigh flap (ALTF) was performed in 11%. In the LMWH group, the proportion of ALTF (32%) to RFF (66%) is significantly higher in comparison to the UFH group (*P* = 0.002). We determined a salvage situation in 6.9% in UFH group and 7.8% in LMWH group (*P* = 0.778) with no significant difference regarding the radiation dose delivered (*P* = 0.244). Both patient groups did not differ significantly with respect to gender (*P* = 0.288), age (*P* = 0.202), ASA score (*P* = 0.250), history of smoking (*P* = 0.582) and alcohol consumption (*P* = 0.449), as well as pre-existing medical conditions (see Table 1 for details). Notably, we observed a larger proportion of NYHA stage II and III patients in the UFH group (*P* = 0.026). The average length of hospitalization was 21.3 ± 9.3 and 20.1 ± 16.3 days with an average stay on intermediate care unit of 5.0 ± 2.6 days and 4.3 ± 1.9 days, respectively, for group UFH and LMWH.

### Postoperative outcome

All outcome parameters are presented in Table 2. Overall, flap survival was 94.4% (251/266). The flap survival rate was 96.6% (84/87) and 93.3% (167/179) for the groups UFH and LMWH, respectively (*P* = 0.280). Three total (3.4%) and five partial (5.7%) flap losses occurred, in group UFH, whereas 12 total (6.7%) and four partial (2.2%) flap losses occurred in group LMWH (*P* = 0.280 and 0.134; Table 2).

Thrombosis of the vascular pedicle was registered, on average, 57.5 ± 86.4 h after surgery and led to immediate surgical exploration. We observed venous pedicle involvement in 5.7% and 8.4% for the groups UFH and LMWH, respectively (*P* = 0.445). Besides, in two cases of the UFH group (2.3%) and two cases of the LMWH group (1.1%), the arterial pedicle was additionally occluded. We observed an isolated occlusion of the arterial pedicle in further three (3.5%) UFH cases and two (1.1%) LMWH cases (*P* = 0.040). In summary, we can confirm a total flap thrombosis rate of 9.2% and 9.5% of the UFH and LMWH group, respectively (*P* = 0.767; Table 2). Reperfusion was achievable in all cases of revision procedures. The logistic regression analysis indicated no superiority of LMWH over UFH concerning the occurrence of flap thrombosis (Odds ratio 1.100; 95% CI 0.469–2.577; *P* = 0.827). The regression analysis of potential confounding variables does not show any significant relation to age (*P* = 0.299), type of flap (*P* = 0.142), number of venous anastomoses (*P* = 0.904), the use of a coupler device (*P* = 0.692), ASA score (*P* = 0.999), number of pack-years (*P* = 0.999), and applied radiation dose before surgery (*P* = 0.684) on pedicle thrombosis. The odds ratio in relation to the operation time was calculated as 0.998 (95% CI 0.995–1.00; *P* = 0.028). A multivariate regression analysis was not assessable due to the low rates of events.

The rate of major postoperative bleeding was 4.6% and 6.7% (*P* = 0.498), the rate of major hematoma requiring evacuation was 4.6% and 3.9% (*P* = 0.792) for the UFH and LMWH group. Major salivary fistulas were found in 4.6% (UFH) and 6.7% (LMWH) of cases (*P* = 0.498). Besides, we did not diagnose any case of heparin-induced thrombocytopenia or allergic reactions. The overall revision rate, including sole neck exploration without revision of the anastomosis, was 20.7% and 20.1% for UFH and LMWH patients, respectively (*P* = 0.912; Table 2). The occurrence of postoperative complications and revision surgery does not seem to significantly compromise the overall survival in this study cohort (68.1% for revision versus 69.2% for no revision; follow-up period of 65.4 ± 42.3 months; *P* = 0.812).

## Discussion

This is the first study comparing low-dose UFH and LMWH in patients with a free microvascular reconstruction of the head and neck. Based on the current data, we can assume comparable eligibility of low-dose, intravenously applied UFH, and subcutaneously applied LMWH. With an overall flap survival rate of 94.4%, the results presented are in line with those of the current literature [12, 15]. We could not find any significant difference regarding the flap survival rate (*P* = 0.280) and flap thrombosis (*P* = 0.767) in both groups (Table 2). Moreover, with an overall revision rate of 20.3%, the results of both therapy groups are also comparable (*P* = 0.912). Revascularization of the pedicle was archived in every case of revision surgery.

In most cases, thrombosis of the vascular pedicle is caused by an interruption of the venous outflow. A thrombus formation in the venous branch, a kink of the pedicle, or the pressure exerted by a hematoma formation are possible causes for venous congestion. Determined by experimental and clinical observations, arterial vessel occlusion occurs, preferably at the site of anastomosis, mostly dependent on fibrin formation [4]. Therefore, anticoagulative drugs seem to improve flap survival by reducing the probability of thrombus formation. Besides, the “ex vivo” irrigation of the transplant is recommended after flap raising [16]. There are several studies concerning the use of anticoagulants in the perioperative management of patients undergoing free microvascular tissue transplantation. However, the efficacy is not finally apparent [11].

Low-dose UFH is used in some reconstructive centers as standard medication for thrombosis prophylaxis. Chalian et al. administered 300 IU heparin/kilogram body weight/day by permanent infusion, confirming a significantly higher thrombosis rate using the external jugular vein compared to the internal jugular vein [17]. Overall, flap survival was 96%. Pohlenz et al. conducted a retrospective study referring to the anticoagulation with 200 IU heparin/kilogram body weight/day via infusion. Even with this lower dosage, a flap survival rate of 97.1% could be achieved [18]. However, the rate of postoperative medical, especially pulmonary and cardiac complications, was 11.4%. In a comparative study, Deutinger et al. examined the superiority of subcutaneous heparin in combination with dextran from intravenous administration of UFH at a dose of 500–800 IU/h as a continuous infusion over 24 h, concerning no significant impact on flap thrombosis (7.4% versus 6.5%; *P* = 0.790) [19]. This dosage of UFH is comparable to that of our study (500 IU/h).

In the regression analysis, we examined many different accompanying factors that could be correlated with flap failure. None showed a significant influence on flap survival. This is in line with an extensive study by Kroll et al. from the 1990s. On 854 patients, it was demonstrated that factors, such as age, smoking, and prior irradiation, in particular, do not affect flap survival [20]. Because venous thrombosis is the most common cause of vascular complications, many surgeons recommend anastomosing two veins [21–23]. Interestingly, in regression analysis, we could not find any advantage of dual or triple venous anastomosis compared to a single-vein anastomosis (*P* = 0.904). The reason for this could be the use of the microvascular coupler in more than 80% of cases because it achieves advantages over the sutured anastomosis even in single-vein anastomosis [24–26]. Despite the use of a microvascular coupler, postoperative thrombosis prophylaxis is indispensable.

LMWH is currently the most commonly used postoperative thrombosis prophylaxis. It is a derivative of unfractionated heparin produced by the determinative hydrolysis of standard heparin into short polysaccharide fragments. These molecules have the same inhibitory effect on the active factor X, but a weaker anti-thrombin activity. The half-life is approximately 4 h [9]. A retrospective analysis by Reiter et al. showed favorable results with a flap survival rate of 97.1% using subcutaneously applied LMWH (20 mg or 40 mg) [12]. A drawback is a lack of information about medical complications and the absence of a control group. Zhou et al. conducted a randomized, prospective trial, determining no significant difference in flap failure (*P* = 0.615) between patients with aspirin and dextran, patients with LMWH, and those who did not receive any antithrombotic medication at all [27]. However, it was remarkable that patients with LMWH significantly more often developed hematomas in need of revision (*P* = 0.032). An et al. showed similar results in a cohort of 101 patients who did not receive anticoagulative therapy after microvascular flap reconstruction. The comparison group included 33 patients receiving UFH (2.500 IU in the operation room and postoperative infusion of 200 IU/h for 7 days). The authors could not find any significant differences in the rate of flap necrosis (15.84% versus 18.18%; *P* = 0.75) and hematoma formation (13.86% versus 15.15%; *P* = 0.66) [28]. However, there is no mentioning of information on the type of pedicle thrombosis, the number of revision operations, and medical complications, such as pulmonary artery embolism.

The development of a hematoma, which can lead to secondary thrombosis due to compression of the vascular pedicle, is a feared complication. In our study cohort, we observed a hematoma rate of 4.6% and 3.9% in patients who received either UFH or LMW (*P* = 0.792; Table 2). These results are also consistent with those of other studies [18]. Besides, Kroll et al. found a hematoma rate of 5.3% in patients who did not receive anticoagulation medication undergoing microvascular free-flap transplantation [29]. Furthermore, there was no cause-and-effect relationship between the use of anticoagulants and flap loss or prevention under the application of low-dose UFH (100–400 IU/h).

We can confirm that the low-dose application of UFH in a dosage of 500 IU/h has no significant impact on coagulation parameters, but showed a tendency for increased APTT (e.g., POD1–3, 42.3 ± 12.6 s) compared to the LMWH group (38.6 ± 10.3 s, *P* = 0.077; Table 3). Regarding the coagulation parameters, we could not find significant differences between patients with and without postoperative bleeding in both prophylaxis groups of our study cohort (Table 4). Concerning postoperative medical complications, we found one patient in the UFH group, receiving pulmonary artery embolism (Table 2). Although this was a single incident, there might be a significant result with an increase in the number of cases. Furthermore, there was no case of deep vein thrombosis.

**Table 4:** Coagulation parameters depending on postoperative bleeding

	Bleeding (major and minor)	Mean ± SD	Statistical comparison of groups yes vs. no *P* value
APTT POD 1-3	Yes	40.2 ± 9.5	0.901
	No	40.6 ± 11.9	
POD 3-5	Yes	38.3 ± 4.6	0.706
	No	39.2 ± 20.1	
POD 8-12	Yes	39.7 ± 10.0	0.520
	No	36.4 ± 9.3	
PT POD 1-3	Yes	78.9 ± 11.8	0.675
	No	77.2 ± 12.5	
POD 3-5	Yes	85.9 ± 13.4	0.973
	No	85.8 ± 12.5	
POD 8-12	Yes	64.5 ± 28.7	0.303
	No	82.3 ± 18.7	

*APTT* activated partial thromboplastin time, *POD* postoperative day, *PT* prothrombin time

The presented results need to be interpreted, considering the retrospective character. However, our cohort included every free-flap reconstruction that fulfilled the inclusion criteria at our department between 2004 and 2017. Thus, two groups of patients with a similar distribution of relevant oncological, medical, and surgical parameters were obtained, which reduces the inevitable selection bias. Therefore, a comparison of postoperative thrombosis prophylaxis was possible. Furthermore, there was no negative control group that could prove the superiority of anticoagulative therapy over patients without postoperative thrombosis prophylaxis. In our opinion, however, this comparison is questionable in a randomized, prospective study, since a malignant disease is always a risk factor for the development of thrombosis. Moreover, oncological tumor resections with microvascular reconstructions are associated with long operating times and represent a sole risk factor. Even if there is no clear statement on the perioperative risk of thrombosis in head and neck patients due to a lack of randomized clinical studies, perioperative thrombosis prophylaxis is recommended according to the German guideline [30]. Determining the effect of anticoagulant medication on flap survival is always critical as the outcome depends on multiple factors. The choice of the appropriate graft, a precise surgical technique, and rigorous postoperative monitoring are the keys to a high success rate in microvascular surgery.

Even if we could not determine a significant superiority of one of the anticoagulants, in general, we recommend postoperative thrombosis prophylaxis. According to the recommendations of the current guideline, priority should be given to LMWH over UFH as LMWH has improved pharmacological properties, a lower risk of side effects, better bioavailability with a longer half-life, and reasonable practicability for once-daily administration [29]. However, it should be noted that therapy monitoring and the detection of overdose by determining the APTT are not possible when applying LMWH. In patients with a high risk of postoperative bleeding, we prefer UFH because it is easier to control due to the possibility of titration, a shorter half-life compared to LMWH, and the possibility of antagonism with protamine. Mainly due to the high risk of hematoma development in the neck, this is of importance since such a complication can cause thrombosis of the vascular pedicle. Patients with pedicel thrombosis also receive UFH via infusion, usually within the APTT range of 50–60 s. In conclusion, we can summarize that the choice of thrombosis prophylaxis is still an individual case decision in high-risk patients as the reconstruction of complex three-dimensional defects of the upper aerodigestive tract using free microvascular grafts is still challenging.

## Conclusion

Based on the present data, we cannot demonstrate the superiority of low-dose UFH or LMWH concerning flap survival and the occurrence of postoperative complications. The results after the application of UFH or LMWH are consistent with those reported in the current literature. More important key factors seem to be the surgical technique and careful postoperative monitoring for at least 5 days, as well as the decision to perform immediate surgical revision in case of any sign of thrombosis, which is crucial for flap survival.
